# The Impact of miRNA Expression on Colon Cancer Severity, Invasiveness, and Localization

**DOI:** 10.3390/cancers17071091

**Published:** 2025-03-25

**Authors:** Constantin-Dan Tâlvan, Elena-Teodora Tâlvan, Călin Ilie Mohor, Liviuța Budișan, Valentin Grecu, Manuela Mihalache, Ioana Berindan Neagoe, Oana Zănoagă, George Călin Oprinca, Adrian Nicolae Cristian

**Affiliations:** 1 Faculty of Medicine, “Lucian Blaga” University of Sibiu, 550169 Sibiu, Romania; talvan_dan@yahoo.com (C.-D.T.); calin.mohor@ulbsibiu.ro (C.I.M.); manuela.mihalache@ulbsibiu.ro (M.M.); georgecalin.oprinca@ulbsibiu.ro (G.C.O.); adrian.cristian@ulbsibiu.ro (A.N.C.); 2 Research Center for Functional Genomic, Biomedicine and Translational Medicine, “Iuliu Hațieganu” University of Medicine and Pharmacy Cluj-Napoca, 400012 Cluj-Napoca, Romania; liviuta.petrisor@umfcluj.ro (L.B.); ioananeagoe29@gmail.com (I.B.N.); oana.zanoaga@umfcluj.ro (O.Z.); 3 Faculty of Engineering, “Lucian Blaga” University of Sibiu, 550025 Sibiu, Romania; valentin.grecu@ulbsibiu.ro

**Keywords:** colon cancer, miRNA, invasiveness, grading, localization, biomarkers

## Abstract

**Background/Objectives:** Colorectal cancer (CRC) is a major global health concern with rising incidence and mortality. MicroRNAs (miRNAs) play key roles in tumor progression, invasion, and metastasis. This study aimed to analyze the expression of miR-101-3p, miR-106a-5p, and miR-326 in CRC patients and assess their association with tumor grading and tissue invasion. **Methods:** A total of 40 CRC patients were included in the study. miRNA expression levels were measured using quantitative reverse transcription PCR (qRT-PCR). Statistical analyses, including regression models, were performed to evaluate correlations between miRNA expression, tumor grade, and invasion patterns. **Results:** MiR-101-3p was significantly downregulated in advanced tumors, while miR-106a-5p expression peaked in Grade 2 CRC. MiR-326 expression was associated with organ metastasis. Regression models confirmed complex interaction patterns among these miRNAs, suggesting their potential role in CRC progression. **Conclusions:** These findings indicate that miR-101-3p, miR-106a-5p, and miR-326 are associated with CRC severity and invasion. Their expression patterns suggest potential utility as biomarkers for early detection and targeted therapies. Further studies with larger cohorts are needed to validate these results and explore their clinical applications.

## Simple Summary

This study explores how specific microRNAs (miR-101-3p, miR-106a-5p, and miR-326) influence colon cancer severity, spread, and location. Researchers analyzed 40 patients and found that miR-101-3p was lower in aggressive cancers, miR-106a-5p peaked in intermediate stages, and miR-326 was linked to organ metastasis. These microRNAs could help in early cancer detection and targeted treatments. This study highlights potential gender and age differences in colon cancer progression and suggests that further research is needed to confirm these findings and their clinical applications.

## 1. Introduction

Colorectal cancer (CRC) remains a significant global health burden, accounting for approximately 9% of all cancer cases worldwide. Its incidence increases with age and varies based on geography and lifestyle factors [1]. In 2022, an estimated 1.14 million new cases were reported, with a nearly equal distribution between proximal (50.8%) and distal (49.2%) colon cancers [2]. Notably, the incidence of CRC has been increasing among individuals under 50 at a rate of 2.1% per year [3], a concerning trend also observed in Europe and North America [4]. The global burden of CRC continues to grow, with approximately 2.17 million cases reported in 2019 and an age-standardized incidence rate of 26.7 per 100,000 people [5]. The highest incidence rates were recorded in North America (63.2% for proximal tumors) and North Africa (61.8% for distal tumors) [2]. Between 1990 and 2019, CRC cases have steadily increased, particularly in middle Socio-Demographic Index (SDI) regions, with a net drift of 2.33% [5]. This growing trend is mirrored in recent global cancer reports, emphasizing the urgent need for targeted preventive strategies [6]. Despite the rising incidence, CRC-related mortality has declined in Western countries due to improved screening and early detection programs [7]. Effective screening strategies, such as colonoscopy and stool-based tests, have been pivotal in reducing mortality rates, yet disparities in screening implementation remain a challenge [8]. Given the increasing incidence among younger populations, early diagnosis and public health interventions are crucial [9]. Strengthening global screening efforts and promoting preventive measures, including dietary and lifestyle modifications, are essential to mitigating the rising burden of CRC [10,11].

The relationship between colon cancer and inflammation is multifaceted, with chronic inflammation playing a significant role in the development and progression of colorectal cancer (CRC) [12,13,14]. Research indicates that inflammatory cytokines and systemic inflammatory responses are closely linked to increased cancer risk and poorer outcomes [15]. The inflammatory response significantly influences colorectal cancer (CRC) by modulating tumor growth, progression, and therapy response through immune cell recruitment, cytokine production, and signaling pathway activation, highlighting the critical role of inflammation in the tumor microenvironment of CRC [14].

Research has demonstrated that microRNAs (miRNAs) are intricately involved in the progression of tumors, encompassing processes such as cellular proliferation, cell cycle regulation, apoptosis, as well as tumor angiogenesis and invasion, and they exert a multifaceted and significant influence on tumorigenesis regulation [16,17]. The identification of specific miRNAs has the potential to facilitate the early diagnosis of malignant cells, and the assessment of variations in their expression profiles may function as a prognostic indicator throughout the disease trajectory or its therapeutic interventions [18]. MiRNAs may act as both diagnostic and prognostic biomarkers, in addition to serving as promising therapeutic targets for colorectal carcinoma [19,20]. Studies have shown that certain miRNAs exhibit lower expression levels in cancerous tissues compared to normal tissues, correlating with tumor aggressiveness and grading [20,21]. Some miRNAs have also been identified as potential biomarkers for predicting recurrence-free survival, suggesting their expression levels may reflect tumor grading and aggressiveness [20]. MiR-101-3p is significantly decreased in colon cancer tissues, indicating a potential loss of its tumor-suppressive functions [22]. Furthermore, in advanced cancer stages, the expression of miR-101-3p further declines, correlating with increased tumor severity [18]. The expression levels of miR-101 were found to be significantly reduced in cases of colon carcinoma. The enhanced expression of miR-101 demonstrated a capacity to suppress cellular proliferation and migratory behavior, while simultaneously augmenting the susceptibility of colon cancer cells to chemotherapeutic agents [22]. The involvement of miR-106a-5p in colorectal carcinoma is intricate, affecting tumor advancement, resistance to chemotherapeutic agents, and its viability as a biomarker. Empirical studies suggest that miR-106a-5p is frequently overexpressed in colorectal cancer (CRC) tissues, exhibiting a significant correlation with unfavorable prognostic outcomes and aggressive phenotypic traits [23]. MiR-106a-5p enhances the proliferation, migration, and invasion of colorectal cancer (CRC) cells through the modulation of DDX5, which subsequently initiates the activation of the AKT signaling cascade [24]. Furthermore, it plays a significant role in the epithelial–mesenchymal transition (EMT), thereby facilitating metastasis via the downregulation of TGFβR2 [25]. Elevated levels of miR-106a-5p have been linked to resistance against 5-fluorouracil (5-FU) in colorectal cancer (CRC) by inhibiting apoptosis and enhancing cell survival. This resistance mechanism underscores the potential of miR-106a-5p as a therapeutic target for overcoming chemoresistance in CRC [25]. MiR-326 plays a complex role in colorectal cancer (CRC), primarily acting as a tumor suppressor by inhibiting cell proliferation, migration, and invasion. Its expression is significantly downregulated in CRC tissues compared to normal tissues, suggesting its potential as a biomarker for CRC [19,26]. MiR-326 directly regulates the nin one binding protein (NOB1), resulting in decreased cell proliferation and enhanced apoptosis in colorectal cancer (CRC) cells, improving prognosis in CRC patients [26]. The overexpression of miR-326 suppresses CRC cell growth by targeting PNO1, a protein essential for proteasome assembly. This inhibition increases the stability of p27, a key regulator of the cell cycle, thereby impeding cancer progression [27].

This study investigates the expression levels of miR-101-3p, miR-106a-5p, and miR-326 in relation to cancer grading and invasion, while also assessing the correlations among these three microRNAs.

## 2. Material and Method

### 2.1. Study Population

The present study included 40 patients diagnosed with colon cancer who underwent surgical intervention at the Regional Institute of Gastroenterology and Hepatology in Cluj-Napoca and the County Emergency Hospital of Sibiu over a 12-month period (see Table 1). The study cohort (n = 40; 22 females, 18 males; age range: 45–86 years) was stratified into three groups based on the grading classification for colon cancer, as follows: Grade 1 group—patients with well-differentiated colon cancer, Grade 2 group—patients with moderate differentiated colon cancer, Grade 3 group—patients with poorly differentiated colon cancer (the study did not include Grade 4—undifferentiated colon cancer patients). Additionally, further analysis divided the patients into five groups according to tissue invasion: submucosal, muscular, serosa, lymphatic invasion, and organ metastasis. Furthermore, we divided patients according to cancer location. MiRNA expression was analyzed between the groups and correlations between the three miRNAs were made for each group.

Prior to enrollment, all participants received a comprehensive explanation of the study objectives and provided written informed consent following a thorough medical evaluation. The inclusion criteria required patients to have a histopathologically confirmed diagnosis of colon adenocarcinoma, an adequate performance status, and no history of prior chemotherapy or radiotherapy. Patients were excluded if they had autoimmune or inflammatory disorders, had undergone treatments known to alter miRNA expression (such as chemotherapy or radiotherapy), had other malignancies or active infections, or had experienced cardiovascular events within the last six months.

The study was approved by the Ethics Committee of the Regional Institute of Gastroenterology and Hepatology in Cluj-Napoca, Romania (approval no. 2769/1.03.2018), the Iuliu Hațieganu University of Medicine and Pharmacy in Cluj-Napoca, Romania (approval no. 155/02.04.2018), and the County Emergency Hospital of Sibiu, Romania (approval no. 10759/23.05.2019).

### 2.2. Sample Collection

The study cohort comprised 40 patients diagnosed with colon adenocarcinoma, confirmed through histopathological examination. For each patient undergoing surgical intervention for colon cancer, two tissue samples were collected intraoperatively: one from the tumor and one from the peritumoral region, the latter being analyzed for microscopic cell infiltration. The collected samples were subsequently processed and preserved in liquid nitrogen at −170 °C to ensure optimal storage conditions.

### 2.3. RNA Analysis

#### 2.3.1. RNA Isolation and Extraction

Total RNA was extracted and isolated from all 40 matched pairs of colon adenocarcinoma samples (tumor and adjacent tissue) using the phenol–chloroform (Tri-Reagent) method, following the manufacturer’s protocol. RNA concentration was assessed using the NanoDrop-1000 spectrophotometer (Thermo Fisher Scientific, Waltham, MA, USA) and was found to be 50 ng/μL across all samples.

#### 2.3.2. cDNA Synthesis and Quantitative Real Time RT-PCR

MiRNA expression levels were analyzed in 40 tumor tissue samples. Following quantification, 50 ng of total RNA was reverse-transcribed into complementary DNA (cDNA) using the TaqMan MicroRNA Transcription Kit (Thermo Fisher Scientific, Waltham, MA, USA) and the TaqMan MicroRNA Primer Assay (Thermo Fisher Scientific, Waltham, MA, USA) for the selected miRNAs (miR-101-3p, miR-106a-5p, and miR-326), in accordance with the manufacturer’s protocol. The primer sequences for the selected miRNAs are as presented in Table 2:

Quantitative reverse transcription polymerase chain reaction (qRT-PCR) was conducted in a total reaction volume of 10 μL, comprising 5 μL of complementary DNA (cDNA) diluted 1:5 with nuclease-free water, 5.03 μL of TaqMan Fast Advanced Master Mix (Applied Biosystems, Waltham, MA, USA), and 0.47 μL of primers specific for each miRNA. The amplification was performed using the ViiA7 PCR system (Applied Biosystems, Waltham, MA, USA) under the following cycling conditions: an initial denaturation step at 50 °C for 2 min and 95 °C for 2 s, followed by 40 cycles of 95 °C for 1 s and 60 °C for 20 s. RNU48 and RNU6B were employed as housekeeping miRNAs for normalization. The relative expression levels were analyzed using the ΔΔCT method. Statistical analysis of the obtained cycle threshold (CT) values was conducted using GraphPad Prism software v.6 (GraphPad Software, San Diego, CA, USA).

### 2.4. Statistical Analysis

The statistical analysis of the data was conducted using a systematic and rigorous approach to ensure accuracy and reliability in interpreting the results. The raw qRT-PCR data were initially curated and structured using Microsoft Excel, where data cleaning was performed to remove inconsistencies and prepare the dataset for further statistical processing. Minitab 20 was used for all statistical analyses, employing both descriptive and inferential methods to explore trends, assess differences, and evaluate relationships between miRNA expression levels and clinical parameters.

Descriptive statistics were used to summarize the distribution of miRNA expression levels, including measures of central tendency (means and medians) and dispersion (standard deviations and interquartile ranges). These statistics provided an overview of the data’s variability and distribution patterns. To visualize these distributions, we generated boxplots and histograms, which helped identify potential outliers and assess whether the data followed a normal distribution.

To compare miRNA expression levels between two independent groups, we applied two-sample *t*-tests, which allowed us to determine whether the mean expression levels differed significantly. This test was appropriate because it assumes normality in each group and equal or unequal variances, which were assessed using Levene’s test for homogeneity of variances.

For comparisons involving more than two groups, one-way ANOVA tests were performed. These analyses assessed whether there were statistically significant differences in miRNA expression levels across multiple tumor grades or tissue invasion stages. When ANOVA indicated significant differences (*p* < 0.05), we conducted post-hoc Tukey’s tests to identify which specific groups differed from each other. One-way ANOVA was selected due to its ability to compare multiple independent groups simultaneously while controlling for Type I error.

In addition to group comparisons, we conducted multiple regression analyses to examine relationships between miR-106a-5p and predictor variables (miR-101-3p and miR-326), while considering tumor grade and tissue invasion stage as potential moderating factors. Interaction terms were included in the regression models to assess whether these associations varied across different stages of tumor progression. We interpreted regression coefficients to determine the direction and strength of these relationships, with statistical significance assessed using *p*-values (*p* < 0.05). The coefficient of determination (R^2^) was also examined to evaluate how well the predictor variables explained the variance in miR-106a-5p expression.

## 3. Results

### 3.1. Demographic Analysis

Table 3 summarizes the age and gender distribution across three colon cancer grades (G1, G2, G3), detailing participant numbers, mean age, variability, and age ranges. Across all grades, males tend to have a higher mean age than females. In Grade G1, the mean age was 56 years for females and 67.8 years for males. In Grade G2, females had a mean age of 67.36 years compared to 70.91 years for males, with the greatest age variability observed in females (StDev = 13.37). In Grade G3, mean ages were 67.33 years for females and 69 years for males. Across all grades, the mean age for males tends to be higher than that for females, indicating a potential gender-related difference in age distribution among colon cancer patients. The median age aligns closely with the mean for most groups, suggesting a relatively symmetric distribution of ages. These data highlight the differences in age and gender distribution, which may be relevant for understanding the demographic patterns associated with colon cancer grading.

Table 4 analyzes age and gender distribution across tissue invasion types in colon cancer. Males generally have a higher mean age than females across all categories, with notable differences in lymphovascular invasion (mean age: 68.60 years for males vs. 62.71 for females) and organ metastasis (80.00 years for males vs. 75.67 for females). Age variability is highest in lymphovascular invasion, while serous invasion shows similar mean ages for both genders. Limited data on submucosal invasion (N = 1 per gender) reveal ages of 56 years for females and 78 years for males. These patterns may reflect demographic trends in cancer progression.

Table 5 shows that males generally have a higher mean age than females for both left-sided and right-sided colon tumors. For left-sided tumors, females had a mean age of 63.56 years, compared to 68.50 years for males. For right-sided tumors, females had a mean age of 68.23 years, while males averaged 71.50 years. Age variability was slightly greater in right-sided tumors for both genders. These patterns may indicate age- and gender-related differences in tumor progression based on location.

**Figure 1:**
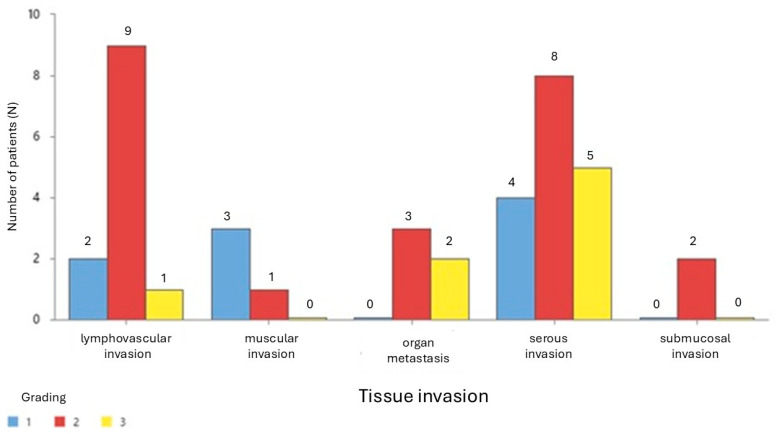
Cancer grading by tissue invasion.

The data in Figure 1 suggest that Grade 2 colon cancer is most frequently associated with tissue invasion across most types, particularly lymphovascular and serous invasion. This finding highlights the aggressive infiltrative nature of intermediate-grade tumors compared to Grades 1 and 3. Grade 1 cases demonstrate higher involvement in muscular invasion, which might indicate a less-advanced disease state with localized spread. Grade 3, although representing the most severe form, has variable involvement across tissue types, with notable cases in serous invasion and organ metastasis. These findings underscore the heterogeneity in invasive patterns across different tumor grades and may guide tailored therapeutic approaches based on cancer grading and tissue infiltration profiles.

The analysis indicated that while females had a slightly higher mean grade (2.1364) compared to males (1.7778), the difference was not statistically significant at the 0.05 level (*p* = 0.088). The 95% confidence interval for the mean difference (−0.0562, 0.7734) included zero, suggesting that the observed variation could be attributed to random fluctuations rather than a systematic difference based on gender. Additionally, the overlapping confidence intervals for both groups further support the lack of statistical significance. These results imply that gender does not play a determining role in grading within the examined sample. Future studies could benefit from a larger sample size or the inclusion of additional factors to better understand potential influences on academic performance.

### 3.2. Descriptive Analysis in miRNA Expression

The analysis of the data presented in Table 6 suggests location-specific differences in miRNA expression within colon cancer, with left-sided tumors exhibiting higher levels of miR-101-3p, miR-106a-5p, and miR-326 compared to right-sided tumors. Among these, miR-106a-5p shows the most pronounced difference, hinting at a potentially greater role in the pathogenesis or progression of left-sided colon cancer. These findings align with the growing recognition of molecular differences between left- and right-sided colon cancers, suggesting that these miRNAs could provide insights into tumor biology and serve as potential biomarkers or therapeutic targets.

However, statistical analysis using a two-sample *t*-test found no significant differences in the expression of miR-101-3p, miR-106a-5p, and miR-326 between tumor locations. The *p*-values for miR-101-3p (0.157), miR-106a-5p (0.293), and miR-326 (0.442) all exceed the 0.05 significance threshold, and the 95% confidence intervals include zero, indicating that the observed expression differences may result from random variation rather than a true biological distinction. Consequently, these miRNAs may not be reliable biomarkers for distinguishing left- and right-sided colon cancers. Future research with larger sample sizes or alternative analytical approaches may provide further clarity on their potential role in tumor biology and clinical applications.

The analysis of miRNA expression across different cancer grades, presented in Table 7, suggests that miR-101-3p, miR-106a-5p, and miR-326 exhibit their highest expression levels in Grade 2 tumors. Specifically, miR-101-3p and miR-326 show a decline in expression in Grade 3, while miR-106a-5p peaks in Grade 2, indicating potential regulatory roles in intermediate-grade tumors. These patterns suggest that the involvement of these miRNAs in tumor biology may be more pronounced in earlier stages but diminishes as cancer progresses to higher grades.

However, statistical validation using one-way ANOVA for each miRNA indicates no significant differences in mean expression across cancer grades. The *p*-values for miR-101-3p (0.700), miR-106a-5p (0.728), and miR-326 (0.460) all exceed the 0.05 threshold, meaning the observed variations in expression levels may be due to random variation rather than a true biological distinction. The means comparison chart further confirms that the confidence intervals overlap, reinforcing the lack of statistically significant differences among the grades.

These findings suggest that, while the descriptive data indicate trends in miRNA expression across tumor grades, there is insufficient statistical evidence to conclude that these miRNAs play a grade-specific role in colon cancer progression. Future studies with larger sample sizes or alternative analytical approaches may be necessary to further investigate their potential significance in cancer grading and therapeutic applications.

Table 8 presents the mean expression levels of miR-101-3p, miR-106a-5p, and miR-326 across different types of tissue invasion in colon cancer. miR-101-3p exhibits the highest expression in muscular invasion (mean = 1.113) and the lowest in submucosal invasion (mean = 0.1282), with a right-skewed distribution. miR-106a-5p is most expressed in lymphovascular invasion (mean = 1.547) but shows lower expression in other invasion types, with notable variability and skewness. miR-326 reaches its peak expression in organ metastasis (mean = 0.637) and shows lower levels in serous and submucosal invasion.

Notably, lymphovascular invasion demonstrates high variability and skewness for all three miRNAs, indicating a potential role in tumor progression. These findings highlight differences in miRNA expression across invasion types, suggesting their involvement in colon cancer progression and their possible relevance as biomarkers or therapeutic targets.

### 3.3. MiRNA Correlations in Cancer Grading and Invasion

**Figure 2:**
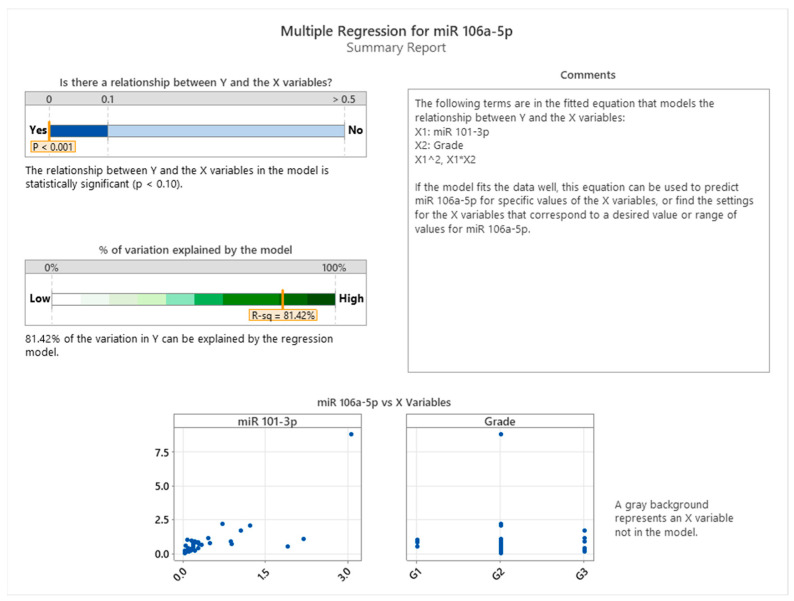
Multiple regression between miR 106-5p, miR-101-3p expression, and tumor grade.

Figure 2 presents the summary results of a multiple regression analysis performed to evaluate the relationship between miR-106a-5p expression (dependent variable) and miR-101-3p expression, while accounting for tumor grade as a factor representing tissue infiltration in colon cancer patients. The analysis indicates a statistically significant relationship between the dependent variable and the predictors included in the model (*p* < 0.001). The model explains 81.42% of the variation in miR-106a-5p expression (R-squared = 81.42%), highlighting a strong predictive power of the selected variables. The fitted regression equation incorporates miR-101-3p (X1), grade (X2), and their interaction (X1*X2), which provides a more nuanced understanding of the interplay between these factors in influencing miR-106a-5p expression. The scatter plots below the summary table illustrate the distribution of miR-106a-5p against miR-101-3p and grade, respectively, showing the patterns of association. These findings underscore the potential regulatory link between miR-101-3p and miR-106a-5p, mediated by tumor infiltration stages, offering valuable insights into miRNA dynamics in colon cancer progression.

**Figure 3:**
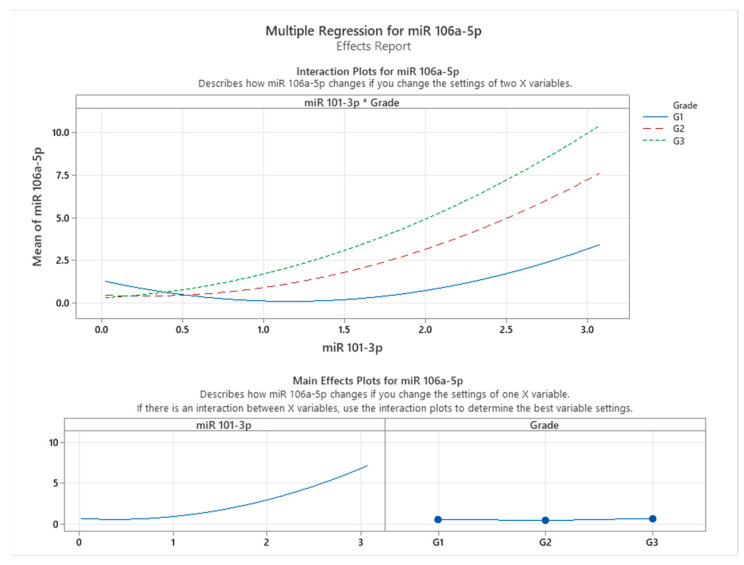
Interaction between miR-101-3p expression and tumor grade (G1, G2, G3) in regulating miR-106a-5p.

Figure 3 illustrates the effects and interaction plots for the multiple regression model assessing the influence of miR-101-3p expression and tumor grade on miR-106a-5p expression in colon cancer patients. The interaction plot at the top highlights how the relationship between miR-101-3p and miR-106a-5p varies across different tumor grades (G1, G2, and G3). A clear interaction effect is observed, as the slope of the relationship between miR-101-3p and miR-106a-5p becomes steeper with increasing tumor grade. Specifically, in patients with Grade G3 tumors, miR-106a-5p expression exhibits the highest sensitivity to changes in miR-101-3p levels, as indicated by the steepest curve, followed by G2 and G1 grades. This finding suggests that the regulatory effect of miR-101-3p on miR-106a-5p intensifies with more advanced tumor infiltration.

The main effects plots shown at the bottom further delineate the individual contributions of miR-101-3p and grade to miR-106a-5p expression. The plot for miR-101-3p demonstrates a non-linear relationship, where miR-106a-5p expression increases exponentially as miR-101-3p levels rise. Meanwhile, the grade plot indicates that higher tumor grades (G2 and G3) are associated with a marked increase in miR-106a-5p expression, reinforcing the hypothesis of tumor grade as a significant modulating factor in this relationship.

These findings indicate a significant correlation between miR-101-3p and miR-106a-5p expression, suggesting a potential association in colon cancer tissues. This relationship appears to vary with tumor grade, highlighting the complex interplay between miRNA expression patterns and cancer progression. While our results do not establish a direct regulatory mechanism, they suggest that miR-106a-5p expression is influenced by tumor characteristics and its association with miR-101-3p. These insights contribute to understanding the molecular dynamics of miRNA interactions in colon cancer and underscore the potential of miR-106a-5p as a biomarker for disease progression. Future studies are needed to determine whether this association has implications for targeted therapeutic strategies addressing miRNA networks at different cancer stages.

**Figure 4:**
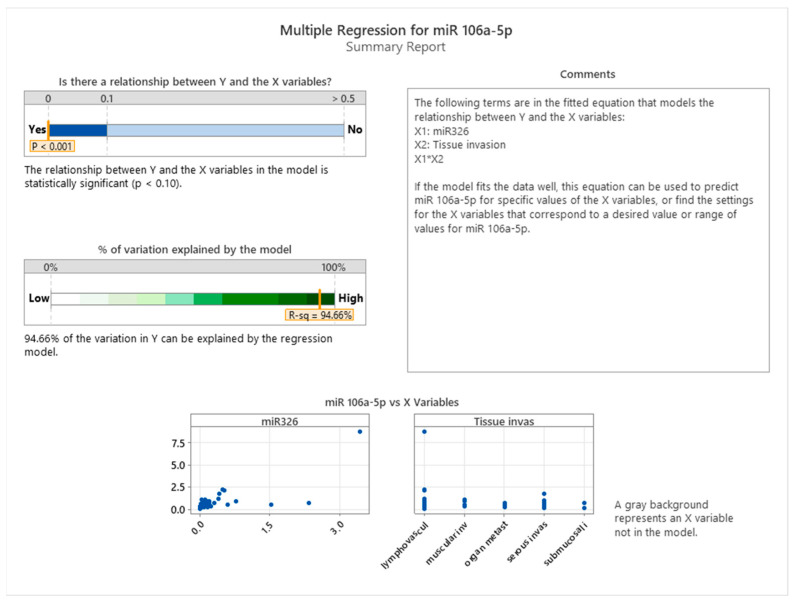
Multiple regression between miR-326, tissue invasion, and miR-106a-5p.

Figure 4 presents the results of a multiple regression analysis evaluating the influence of miR-326 expression and tissue invasion stage on miR-106a-5p expression. The model demonstrates a statistically significant relationship between the dependent variable (miR-106a-5p) and the predictors (miR-326 and tissue invasion), as evidenced by a *p*-value of less than 0.001. The regression model explains 94.66% of the variation in miR-106a-5p expression (R-squared = 94.66%), indicating a robust predictive capability of the included variables.

The fitted regression equation incorporates miR-326 (X1), tissue invasion (X2), and their interaction term (X1*X2), signifying that the relationship between miR-326 and miR-106a-5p is modulated by the level of tissue invasion. The scatter plots at the bottom provide insights into the relationships among these variables. The plot on the left shows a non-linear trend, suggesting that higher levels of miR-326 expression are associated with increased miR-106a-5p levels. The plot on the right indicates distinct patterns in miR-106a-5p expression across different tissue invasion stages, with the highest expression observed in cases of serosa invasion.

These findings highlight a significant association between miR-326 and miR-106a-5p expression, particularly in relation to tissue invasion. However, this study does not establish a causal relationship between these miRNAs. The observed association varies across different stages of tissue invasion, with a stronger correlation in more advanced stages. This suggests that miR-326 and miR-106a-5p may be influenced by similar biological factors during invasive cancer progression. The high explanatory power of the model (94.66%) underscores the importance of these miRNAs in colon cancer dynamics, offering potential insights for stage-specific therapeutic research.

**Figure 5:**
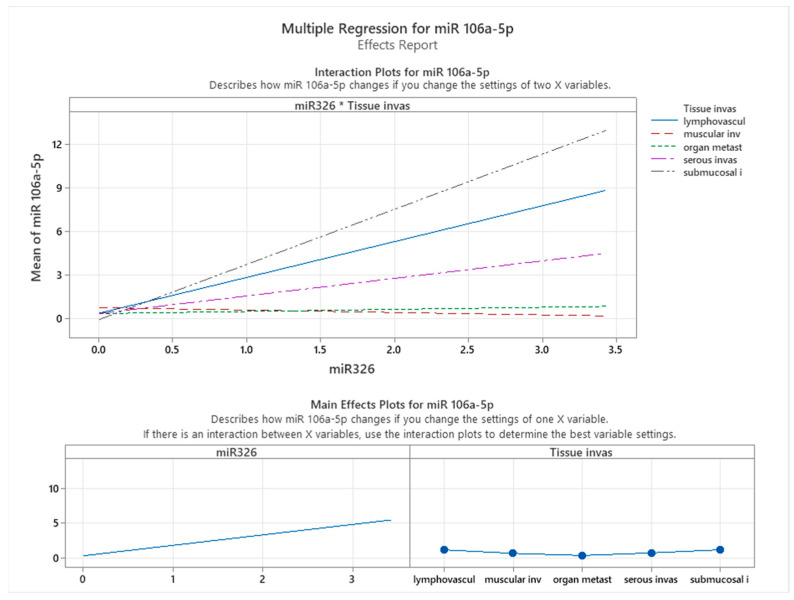
Effects of miR-326 and tissue infiltration on the expression levels of miR-106a-5p.

Figure 5 illustrates the interaction and main effects of miR-326 and tissue infiltration on the expression levels of miR-106a-5p, as assessed through multiple regression analysis. The interaction plot (top panel) highlights how the relationship between miR-326 and miR-106a-5p expression is modulated by different tissue invasion types. The varying slopes of the regression lines across tissue categories suggest that miR-106a-5p expression responds differently to miR-326 levels depending on the invasive environment. Notably, lymphovascular invasion and muscular invasion exhibit the steepest slopes, indicating a stronger positive correlation between miR-326 and miR-106a-5p expression. In contrast, organ metastasis, serous invasion, and submucosal invasion display flatter slopes, suggesting a weaker association between these two miRNAs in these contexts.

This differential expression pattern suggests that miR-326 levels are associated with miR-106a-5p expression, particularly in more aggressive invasion patterns, such as lymphovascular and muscular invasion. The variations in expression across different tissue invasion types indicate that additional biological factors may influence the relationship between these miRNAs. In submucosal and serous invasion, distinct expression dynamics suggest that alternative mechanisms may be at play, potentially affecting the observed association between miR-326 and miR-106a-5p.

The main effects plot (bottom panel) further corroborates these observations. A positive association between miR-326 and miR-106a-5p expression is consistently observed across the dataset. However, the variation in tissue invasion effects suggests that miR-106a-5p expression is influenced by multiple factors, including the invasion context. Among the tissue types analyzed, lymphovascular invasion exhibits the highest mean expression levels of miR-106a-5p, highlighting its potential involvement in vascular dissemination and metastatic progression.

These findings underscore the complex interplay between miRNA expression and the tumor microenvironment, emphasizing the necessity of context-specific analyses when investigating miRNA regulatory networks in colon cancer. The observed interaction between miR-326 and tissue-specific characteristics in association with miR-106a-5p expression suggests that these miRNAs may serve as potential biomarkers or therapeutic targets, particularly in invasion pathways associated with high metastatic risk.

**Figure 6:**
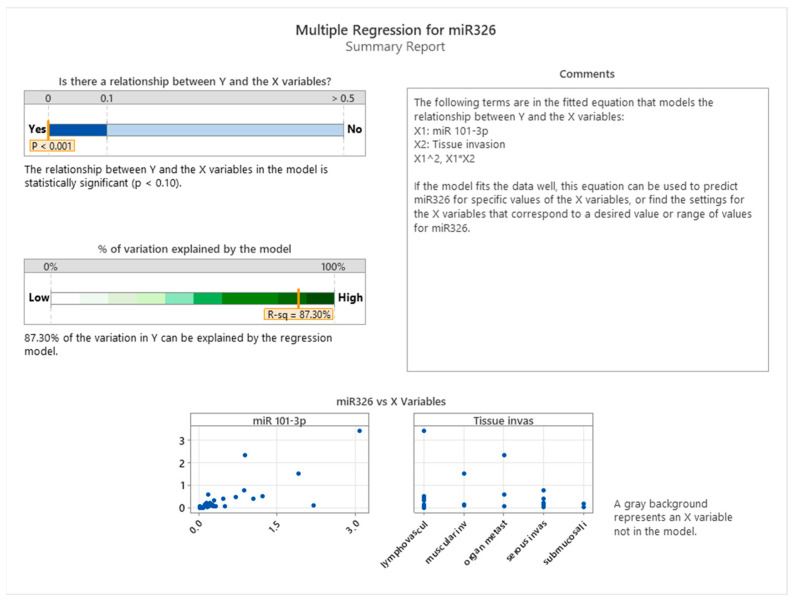
Multiple regression analysis of miR-326 expression with miR-101-3p and tissue invasion as predictors.

Figure 6 summarizes the results of a multiple regression analysis aimed at understanding the relationship between miR-326 expression (Y) and two independent variables, miR-101-3p (X1) and tissue invasion (X2), including their interaction terms (X1^2^ and X1*X2). The analysis reveals a statistically significant relationship between miR-326 and the predictors included in the model, as indicated by a *p*-value < 0.001. This finding suggests that the model reliably explains variation in miR-326 expression based on the given predictors.

The regression model accounts for 87.30% of the variation in miR-326 expression, as demonstrated by the R-squared value, which is notably high. This indicates that the majority of the variance in miR-326 expression can be attributed to the combined effects of miR-101-3p, tissue invasion, and their interactions. The high explanatory power of the model underscores the strong relationship between these variables and miR-326 expression in the studied context of colon cancer.

The scatterplots in the bottom panel provide a visual representation of the relationship between miR-326 and its predictors. The scatterplot for miR-101-3p suggests a positive association, as higher levels of miR-101-3p correspond to increased expression of miR-326. However, the distribution of points appears to widen at higher values of miR-101-3p, which may indicate some degree of variability in this relationship that could be influenced by additional factors, such as tissue-specific characteristics. The scatterplot for tissue invasion reveals distinct clustering of data points based on the invasion type, suggesting that miR-326 expression varies according to the type of tissue infiltration. This further supports the hypothesis that tissue-specific factors play a crucial role in modulating miRNA expression in colon cancer.

**Figure 7:**
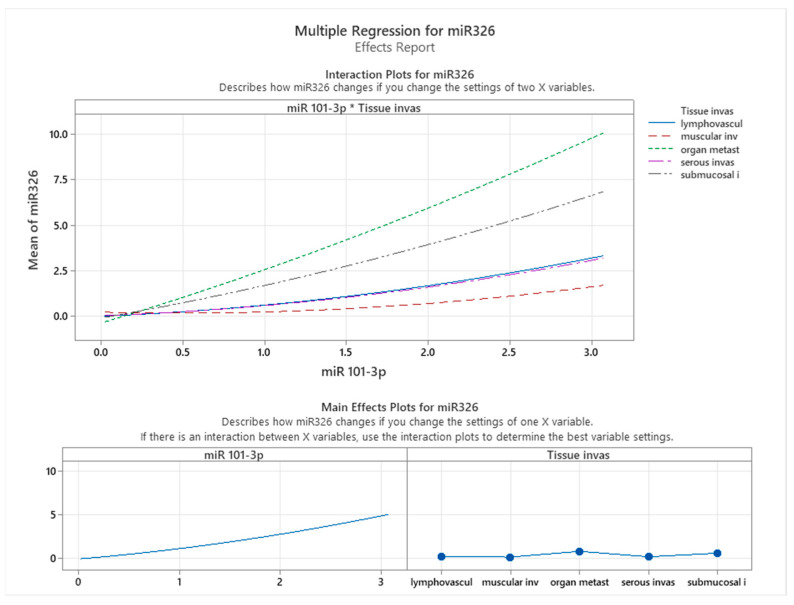
Effects of miR-101-3p and tissue infiltration on the expression levels of miR-326.

Figure 7 presents the interaction and main effects of miR-101-3p and tissue invasion on the expression levels of miR-326, as derived from multiple regression analysis. The interaction plot reveals that the relationship between miR-101-3p and miR-326 expression is influenced by the type of tissue invasion. Specifically, the slope of miR-101-3p varies across different invasion types, with organ metastasis showing the steepest increase in miR-326 expression as miR-101-3p levels rise. This trend suggests that miR-326 expression is highly sensitive to changes in miR-101-3p in the context of organ metastasis, while other tissue types, such as submucosal invasion, show a much weaker or negligible association.

The main effects plot further confirms that miR-101-3p positively influences miR-326 expression in a consistent manner across all conditions, albeit with varying degrees depending on tissue invasion type. Tissue invasion itself also exhibits variability in its impact on miR-326 expression. Organ metastasis emerges as the invasion type most strongly associated with higher miR-326 levels, followed by lymphovascular invasion, while muscular and submucosal invasion show minimal effects. This underscores the differential regulatory impact of tissue invasion types on miRNA expression.

These findings have important implications for understanding the molecular mechanisms underlying colon cancer progression. The strong interaction between miR-101-3p and miR-326, particularly in the context of organ metastasis, suggests a potential cooperative role of these miRNAs in promoting tumor aggressiveness and metastatic behavior. Moreover, the tissue-specific variation in miR-326 expression highlights the critical influence of the tumor microenvironment on miRNA dynamics. These results emphasize the need for further exploration of miR-101-3p and miR-326 as potential biomarkers or therapeutic targets, especially in advanced stages of colon cancer characterized by organ metastasis. This could aid in the development of precision medicine strategies tailored to specific invasion patterns.

**Figure 8:**
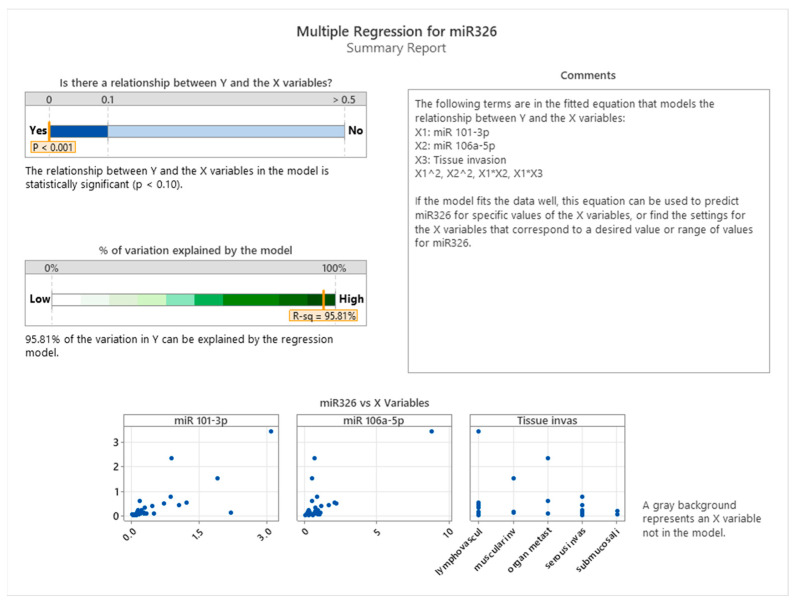
Multiple regression between miR-101-3p, miR-106a-5p, tissue invasiveness, and miR-326.

The summary of the regression model presented in Figure 8 demonstrated a statistically significant relationship between miR-326 and the predictors (*p* < 0.001). This model accounted for 95.81% of the variation in miR-326 expression, as indicated by the high R-squared value. The fitted model included main effects for miR-101-3p, miR-106a-5p, and tissue invasiveness, as well as interaction terms (e.g., miR-101-3p × tissue invasiveness) and non-linear effects (e.g., miR-101-3p squared). This comprehensive approach captures both direct and interactive influences of the predictors.

Scatterplots of miR-326 versus each predictor further illustrate the relationships. For miR-101-3p and miR-106a-5p, a non-linear pattern is evident, supporting the inclusion of quadratic terms in the model. Tissue invasiveness categories, on the other hand, display a categorical influence on miR-326 expression, highlighting the tumor microenvironment’s distinct role in shaping miRNA regulation.

**Figure 9:**
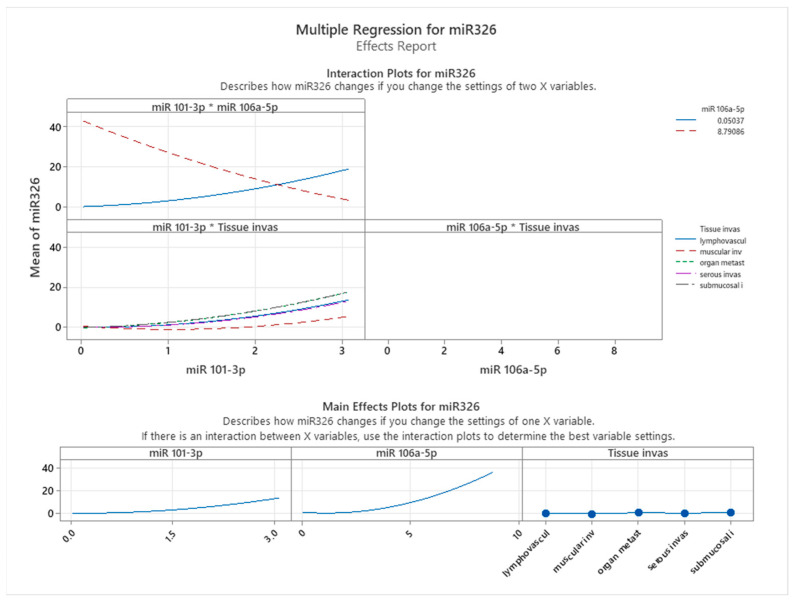
Effects of miR-101-3p, miR-106a-5p, and tissue invasiveness on miR-326.

To understand these relationships further, interaction and main effects plots were examined (see Figure 9). Interaction plots revealed notable effects of predictor interactions on miR-326 expression. Specifically, the interplay between miR-101-3p and miR-106a-5p displayed a dynamic relationship: at lower miR-101-3p levels, higher miR-106a-5p levels correlated with reduced miR-326 expression. However, this relationship reversed at higher miR-101-3p levels, suggesting a context-dependent regulatory effect. The interaction between miR-106a-5p and tissue invasiveness consistently showed that increasing miR-106a-5p levels were associated with elevated miR-326 expression across tissue types. However, the magnitude of this effect varied, with tissue categories such as lymphovascular invasion and organ metastasis exhibiting the highest levels of miR-326 expression. Main effects plots supported these observations. miR-106a-5p exerted a strong positive influence on miR-326 expression, while miR-101-3p demonstrated a moderate, non-linear effect. Tissue invasiveness categories displayed unique mean levels of miR-326 expression, emphasizing the importance of the tumor environment in regulating miRNA levels.

**Figure 10:**
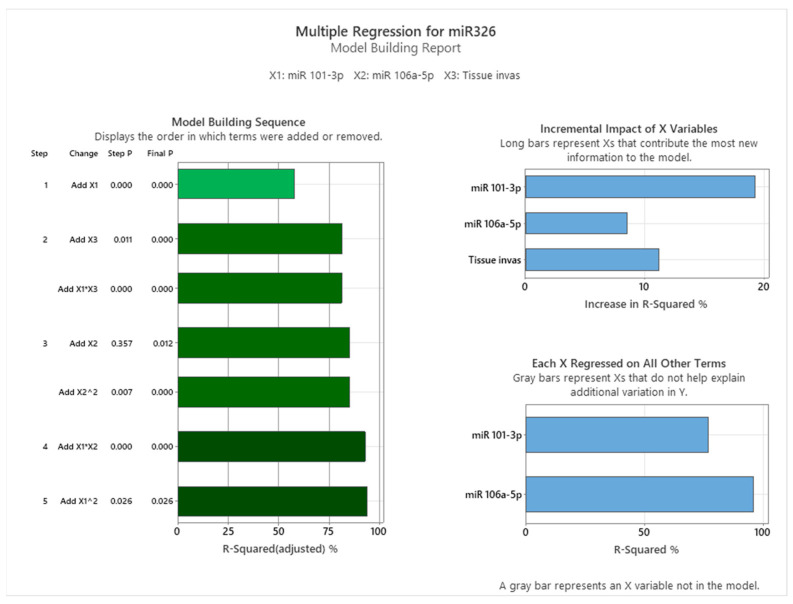
Model Building Analysis.

The stepwise regression approach, as detailed in Figure 10, provided further insights into the incremental contributions of predictors and interaction terms to the model. miR-101-3p was the most significant predictor, contributing the largest increase in adjusted R-squared (~20%), with a *p*-value < 0.001. Tissue invasiveness added an additional ~10% to the explained variance, highlighting its contextual relevance.

While miR-106a-5p had a smaller direct impact, its interaction and non-linear terms significantly enhanced the model fit. For instance, the interaction term between miR-101-3p and tissue invasiveness (X1 × X3) was highly significant, underscoring the complex interplay between these variables. Additionally, quadratic terms for both miRNAs improved the model, further supporting the non-linear relationships observed in the scatterplots.

#### Summary of Findings

Together, these findings highlight the complex nature of miRNA interactions in colon cancer tissues. miR-101-3p expression showed a significant association with miR-326 expression, while miR-106a-5p displayed complex interaction patterns with other miRNAs. These findings indicate correlations rather than direct regulatory mechanisms. Tissue invasiveness emerged as an important contextual factor, influencing miR-326 expression levels in a manner consistent with tumor aggressiveness and stage of invasion. The model explains nearly all observed variation in miR-326 expression, emphasizing the intricate relationships between miRNAs and tumor progression while underscoring the need for further experimental validation.

## 4. Discussion

The present study provides a comprehensive analysis of the expression patterns of miR-101-3p, miR-106a-5p, and miR-326 in colon cancer and their correlation with tumor grading, invasiveness, and localization. The findings contribute to the growing body of literature supporting the role of miRNAs as potential diagnostic and prognostic biomarkers in colorectal cancer (CRC) [20]. Our results align with previous studies that have highlighted the differential expression of these miRNAs in relation to cancer progression and aggressiveness [19].

The findings in our study indicate a consistent trend of higher mean age among male colon cancer patients across all grades (G1, G2, G3), suggesting a gender-related difference in disease onset. This difference is most pronounced in G1, where males exhibit a significantly higher mean age (67.8 years) than females (56 years), aligning with research suggesting delayed onset in men due to genetic and hormonal factors [28,29]. In G2, males still show a higher mean age (70.91 years vs. 67.36 years in females), with the greatest age variability observed among females, potentially influenced by lifestyle and genetic predispositions [10]. In G3, the age gap narrows (69 years in males vs. 67.33 years in females), consistent with studies indicating that aggressive colon cancer affects both genders at similar ages due to accumulated risk factors [30]. These insights highlight demographic variations that may inform tailored screening and prevention strategies. Analysis of age and gender distribution across tissue invasion types further supports a higher mean age in males across most categories, with notable differences in lymphovascular invasion (68.60 vs. 62.71 years) and organ metastasis (80.00 vs. 75.67 years), consistent with studies linking older age with more advanced invasion stages [31]. The greatest age variability in lymphovascular invasion may reflect differential risk factor exposure, while similar mean ages in serous invasion suggest comparable progression patterns [32]. Limited data on submucosal invasion indicate a substantial age gap, highlighting potential early-onset variations. These findings underscore demographic trends in cancer progression and emphasize the need for age- and gender-tailored screening and treatment approaches. Further, age and gender distribution across tumor locations show a higher mean age in males for both left-sided (68.50 vs. 63.56 years) and right-sided (71.50 vs. 68.23 years) colon cancer, reinforcing previous findings that older males may be more susceptible to tumor progression at later stages [33]. Slightly greater age variability in right-sided tumors for both genders aligns with research suggesting distinct biological and molecular characteristics between tumor locations [34]. These findings emphasize the importance of considering tumor location in colorectal cancer screening and management. Analysis of tissue invasion by cancer grade indicates that Grade 2 colon cancer is most frequently associated with invasive patterns, particularly in lymphovascular and serous invasion, emphasizing its aggressive nature [35]. Grade 1 tumors show a higher presence in muscular invasion, possibly reflecting localized spread at an early stage, aligning with findings on early tumor progression [36]. Grade 3 tumors display variable invasion profiles, with notable cases in serous invasion and organ metastasis, consistent with literature on the heterogeneous nature of advanced-stage tumors [37]. These findings highlight the importance of considering tumor grade when evaluating invasion patterns and tailoring treatment strategies accordingly. Figure 2 suggests that while females have a slightly higher mean grade than males, the difference is not statistically significant at the 0.05 level. The 95% confidence interval for the mean difference includes zero, emphasizing that the observed variation may be due to random chance rather than a true difference in cancer grading between genders. These findings align with previous studies indicating that gender does not play a major role in determining colon cancer grade [38].

MiR-101-3p is a crucial regulator in colon cancer progression, acting as a key tumor suppressor that modulates various oncogenic pathways. Its significant downregulation in colorectal carcinoma tissues and cell lines suggests a pivotal role in inhibiting tumor development. Overexpression of miR-101-3p has been shown to suppress cancer cell proliferation, migration, and invasion by directly targeting oncogenic transcription factors such as CREB1, thereby disrupting tumor-promoting signaling pathways [39]. Moreover, miR-101-3p plays a critical role in overcoming therapy resistance, as it counteracts the tumor-enhancing effects of MALAT1, a long noncoding RNA associated with increased survival and radioresistance in colorectal cancer cells [40]. Its involvement in a broader miRNA regulatory network further underscores its potential as a biomarker for early diagnosis and prognosis, as well as a promising therapeutic target for controlling cancer progression [18]. Given its ability to modulate multiple oncogenic mechanisms, restoring miR-101-3p expression may represent a novel strategy for improving treatment outcomes in colon cancer. MiR106a-5p has been found to be overexpressed in adjacent healthy tissues but significantly downregulated in advanced colon cancer stages, suggesting its involvement in early tumor suppression and later dysregulation during cancer progression [18]. Additionally, miR-106a-5p contributes to colorectal cancer metastasis by targeting TGFβR2, thereby promoting epithelial-to-mesenchymal transition [25]. It also interacts with circular RNAs, such as circEGFR, to enhance tumor cell proliferation and invasion through the AKT signaling pathway [24]. These findings indicate that miR-106a-5p may serve as a potential biomarker for early detection and prognosis while also representing a promising therapeutic target for managing metastasis and treatment resistance in colon cancer. MiR 326 downregulation in colorectal carcinoma has been associated with increased tumor aggressiveness, poor prognosis, and enhanced invasion potential [41]. MiR-326 exerts its tumor-suppressive effects by targeting E2F1, a transcription factor involved in cell proliferation and survival, thereby inhibiting cancer cell viability and metastatic potential [41]. Furthermore, miR-326 has been implicated in modulating epithelial–mesenchymal transition (EMT), a process that promotes tumor invasiveness, making it a crucial factor in disease progression [18]. Given its regulatory influence on multiple oncogenic pathways, miR-326 represents a promising biomarker for colon cancer prognosis and a potential therapeutic target for inhibiting tumor invasion and metastasis.

Our results highlight miRNA expression differences in colon cancer based on tumor location, with left-sided cancers exhibiting higher levels of miR-101-3p, miR-106a-5p, and miR-326 than right-sided cancers. miR-106a-5p shows the largest discrepancy, suggesting a significant role in left-sided tumor progression. These findings support growing evidence of molecular distinctions between left- and right-sided colon cancers [42]. The data in our study demonstrate that miR-101-3p, miR-106a-5p, and miR-326 show peak expression in Grade 2 cancers, suggesting a key role in intermediate tumor progression. The decline in miR-101-3p and miR-326 expression in Grade 3 implies reduced regulatory influence, while the marked peak of miR-106a-5p in Grade 2 highlights its potential role in aggressive intermediate-grade tumors. These findings reinforce the importance of miRNA profiling in tumor grading and progression in colon cancer [43,44,45]. Our further findings highlight distinct expression patterns of miR-101-3p, miR-106a-5p, and miR-326 across different types of tissue invasion in colon cancer. miR-101-3p is primarily associated with muscular invasion, miR-106a-5p shows the highest expression in lymphovascular invasion, and miR-326 is most elevated in organ metastasis and submucosal invasion. These findings suggest that each miRNA plays a specific role in regulating tumor invasion, reinforcing their potential as therapeutic targets in colon cancer [18,46].

The correlation between miR-101-3p, miR-106a-5p, and miR-326 with cancer severity has been highlighted in multiple studies. These miRNAs exhibit altered expression patterns in colon cancer, with significant downregulation in advanced stages, suggesting a role in tumor progression. Notably, miR-106a-5p and miR-326 have been strongly associated with cancer severity, indicating their potential utility in prognosis and early detection [18]. Additionally, miR-101-3p has been implicated in cancer progression through its regulatory effects on oncogenic pathways, suggesting its role as a tumor suppressor [47]. These findings emphasize the need for further investigation into the cooperative role of these miRNAs in regulating cancer severity and their potential as biomarkers for tumor progression. The analysis of a multiple regression analysis evaluating miR-106a-5p expression in relation to miR-101-3p and tumor grade in colon cancer demonstrates a statistically significant relationship (*p* < 0.001), with the model explaining 81.42% of the variation in miR-106a-5p expression (R-squared = 81.42%). The regression equation includes miR-101-3p, tumor grade, and their interaction, highlighting a strong predictive interplay [18,48]. The findings suggest that miR-101-3p is positively correlated with miR-106a-5p expression, with tumor grade modulating this relationship. This interaction underscores the role of these miRNAs in tumor progression, particularly in tissue infiltration stages, offering insights into their regulatory dynamics in colon cancer [49]. The multiple regression analysis evaluating the relationship between miR-326, tissue invasion, and miR-106a-5p expression in colon cancer explains 94.66% of the variation in miR-106a-5p expression (R-squared = 94.66%), indicating a strong predictive capability. The interaction plot shows that the association between miR-326 and miR-106a-5p is most pronounced in cases of lymphovascular and muscular invasion, suggesting a potential link to more aggressive invasion patterns. These findings emphasize the relevance of miR-326 and miR-106a-5p expression in the context of tissue invasion and highlight the need for further studies to explore the underlying biological mechanisms [15,23,26]. The multiple regression analysis evaluating miR-326 expression (Y) with miR-101-3p (X1), tissue invasion (X2), and their interactions (X1^2^, X1*X2) as predictors reveals a statistically significant relationship (*p* < 0.001) and explains 87.30% of the variation in miR-326 expression (R-squared = 87.30%). This indicates a strong influence of miR-101-3p, tissue invasion, and their interactions on miR-326 levels in colon cancer. The interaction analysis reveals that miR-326 expression is significantly influenced by tissue invasion type, with organ metastasis demonstrating the steepest increase, while submucosal invasion exhibits a weaker association. Multiple regression analysis evaluating miR-326 expression (Y) with miR-101-3p (X1), miR-106a-5p (X2), tissue invasion (X3), and their interactions (X1^2^, X2^2^, X1X2, X1X3) as predictors reveals a statistically significant relationship (*p* < 0.001) and explains 95.81% of the variation in miR-326 expression (R-squared = 95.81%). This high explanatory power underscores the strong influence of miR-101-3p, miR-106a-5p, tissue invasion, and their interactions on miR-326 levels in colon cancer. The interaction analysis reveals that miR-326 expression is significantly influenced by tissue invasion type, with organ metastasis demonstrating the steepest increase, while submucosal invasion exhibits a weaker association. These findings highlight a strong association between miR-101-3p and miR-326, particularly in aggressive cancer types, suggesting their potential involvement in tumor progression and their relevance as possible therapeutic targets.

Our analysis of miR-101-3p, miR-106a-5p, and miR-326 underscores their crucial roles in tumor suppression, metastasis, and treatment response. The strong correlations between these miRNAs and cancer grading, tissue invasion, and tumor location emphasize their potential as biomarkers for early detection and prognosis. Additionally, our regression models reveal significant interactions among these miRNAs, suggesting complex regulatory networks that influence cancer severity. These findings contribute to a growing body of research supporting miRNA-based diagnostics and therapeutic strategies in colon cancer, warranting further investigation into their clinical applications.

## 5. Study Limitations

This study was conducted on a relatively small cohort (n = 40), which may limit the generalizability of the findings. A larger sample size is needed to validate the observed trends in miRNA expression and cancer grading. While the study establishes correlations between miRNA expression and colon cancer severity, it does not explore the mechanistic roles of miR-101-3p, miR-106a-5p, and miR-326 through functional assays, such as knockdown or overexpression experiments. The research was conducted in a single clinical setting, which may introduce institutional biases. Multi-center studies would enhance the robustness and applicability of the results. This study does not incorporate follow-up data on patient outcomes, limiting insights into the prognostic value of the identified miRNAs in disease progression and treatment response. This study does not account for additional clinical variables such as comorbidities, lifestyle factors, or genetic predispositions that may influence miRNA expression and cancer progression. While miRNA expression was analyzed across different invasion types, the sample size within subcategories (e.g., submucosal invasion) was small, affecting the statistical power of comparisons. Although associations between miRNA expression and colon cancer were identified, the study does not include pathway enrichment analysis to determine the downstream biological effects of miRNA dysregulation.

While our study identifies significant correlations between miR-101-3p, miR-106a-5p, and miR-326, it does not provide functional evidence for direct regulatory interactions. Future experimental studies, such as knockdown or overexpression assays, are necessary to validate potential mechanistic relationships.

These limitations suggest directions for future research, including larger, multi-center cohorts, functional validation studies, and integration of longitudinal patient data to further elucidate the role of miRNAs in colon cancer progression.

## 6. Conclusions

This study provides a comprehensive analysis of the roles of miR-101-3p, miR-106a-5p, and miR-326 in colon cancer progression, grading, and tissue invasion. Our findings highlight a consistent trend of higher mean age among male patients across tumor grades, reinforcing potential gender-related differences in disease onset and progression. Additionally, tumor location-specific variations in miRNA expression suggest that molecular differences between left- and right-sided colon cancers may influence tumor biology and prognosis.

MiR-101-3p was identified as a critical tumor suppressor, significantly downregulated in advanced stages, and associated with a decline in regulatory influence as cancer progresses. MiR-106a-5p exhibited a distinct pattern, peaking in intermediate-grade tumors and playing a pivotal role in promoting epithelial-to-mesenchymal transition and metastasis. Meanwhile, miR-326 demonstrated strong associations with tissue invasion and organ metastasis, reinforcing its function as a regulatory factor in tumor progression. Our multiple regression models confirmed significant interactions between these miRNAs, indicating complex expression patterns associated with colon cancer severity.

The strong correlation between miRNA expression patterns and cancer grading underscores their potential as biomarkers for early detection, prognosis, and therapeutic targets. These findings contribute to the growing body of research supporting miRNA-based precision medicine in colorectal cancer. Future studies should focus on validating these results in larger cohorts, exploring the functional mechanisms underlying these miRNA interactions, and assessing their clinical applicability in personalized treatment strategies.

## Figures and Tables

**Table 1:** Clinicopathological characteristics of subjects.

1. Mean age (years)		67.9 (11.52) ^1^
2. Gender	Male	18 (45%)
	Female	22 (55%)
3. Tumor histology	Adenocarcinoma	40 (100%)
4. Tumor TNM stage	1	5 (12.5%)
	2	16 (40%)
	3	14 (35%)
	4	5 (12.5%)
5. Tumor WHO grade	1	9 (22.5%)
	2	24 (60%)
	3	7 (17.5%)
6. Tumor location	Right colon	21 (52.5%)
	Left colon	19 (47.5%)

^1^—Mean (standard deviation).

**Table 2:** The stem–loop sequences for the selected miRNAs are as follows.

No	miRNAs	Sequence
1	RNU48	GATGACCCCAGGTAACTCTGAGTGTGTCGCTGATGCCATCACCGCAGCGCTCTGACC
2	RNU6B	CGCAAGGATGACACGCAAATTCGTGAAGCGTTCCATATTTTT
3	hsa-miR-101-3p	UACAGUACUGUGAUAACUGAA
4	hsa-miR-106a-5p	AAAAGUGCUUACAGUGCAGGUAG
5	hsa-miR-326	CCUCUGGGCCCUUCCUCCAG

**Table 3:** Age and gender distribution in colon cancer grading.

Grade	Gender	N	Mean	SE Mean	StDev	Variance	Minimum	Median	Maximum
G1	F	2	56.00	9.00	12.73	162.00	47.00	56.00	65.00
	M	5	67.80	2.48	5.54	30.70	62.00	67.00	77.00
G2	F	14	67.36	3.57	13.37	178.86	48.00	68.00	85.00
	M	11	70.91	3.87	12.83	164.69	45.00	74.00	86.00
G3	F	6	67.33	4.35	10.65	113.47	56.00	64.50	84.00
	M	2	69.00	7.00	9.90	98.00	62.00	69.00	76.00

**Table 4:** Age and gender distribution in colon cancer infiltration.

Tissue Invasion	Gender	N	Mean	SE Mean	StDev	Variance	Minimum	Median	Maximum
Lymphovascular invasion	F	7	62.71	4.54	12.01	144.24	48.00	64.00	85.00
	M	5	68.60	6.68	14.94	223.30	45.00	68.00	82.00
Muscular invasion	F	2	62.50	15.50	21.90	480.50	47.00	62.50	78.00
	M	2	63.50	3.50	4.95	24.50	60.00	63.50	67.00
Organ metastasis	F	3	75.67	5.61	9.71	94.33	65.00	78.00	84.00
	M	2	80.00	6.00	8.49	72.00	74.00	80.00	86.00
Serous invasion	F	9	68.00	4.15	12.45	155.00	51.00	69.00	84.00
	M	8	68.63	3.10	8.78	77.13	59.00	65.00	83.00
Submucosal invasion	F	1	56.00	*	*	*	56.00	56.00	56.00
	M	1	78.00	*	*	*	78.00	78.00	78.00

Notes: Values marked with * indicate missing or unavailable data for the respective cell. Each row corresponds to a specific tissue invasion category, further divided by gender (F: Female, M: Male).

**Table 5:** Age and gender distribution in colon cancer location.

Location of tumor	Gender	N	Mean	SE Mean	StDev	Variance	Minimum	Median	Maximum
Left	F	9	63.56	4.23	12.70	161.28	47.00	58.00	84.00
	M	10	68.50	2.70	8.54	72.94	59.00	66.50	83.00
Right	F	13	68.23	3.47	12.52	156.86	48.00	67.00	85.00
	M	8	71.50	4.64	13.13	172.29	45.00	76.50	86.00

**Table 6:** Mean expression of miRNA in colon cancer location.

Variable	Location	N	Mean	SE Mean	StDev	Minimum	Q1	Median	Q3	Maximum
miR 101-3p	Left	19	0.592	0.201	0.876	0.040	0.097	0.181	0.867	3.073
	Right	21	0.2824	0.0628	0.2877	0.0234	0.0908	0.1528	0.3984	1.0513
miR 106a-5p	Left	19	1.124	0.437	1.907	0.216	0.384	0.612	0.956	8.791
	Right	21	0.634	0.119	0.547	0.050	0.242	0.444	0.858	2.217
miR326	Left	19	0.427	0.188	0.817	0.015	0.053	0.097	0.524	3.429
	Right	21	0.258	0.108	0.497	0.006	0.055	0.084	0.281	2.338

**Table 7:** Mean expression of miRNA in cancer grading.

Variable	Grade	N	Mean	SE Mean	StDev	Minimum	Q1	Median	Q3	Maximum
miR 101-3p	1	9	0.391	0.194	0.582	0.047	0.098	0.216	0.381	1.904
	2	23	0.484	0.159	0.763	0.023	0.083	0.147	0.712	3.073
	3	8	0.315	0.113	0.321	0.063	0.146	0.184	0.418	1.051
miR 106a-5p	1	9	0.7303	0.0968	0.2904	0.2159	0.4496	0.8408	0.9324	1.0571
	2	23	0.995	0.372	1.785	0.050	0.264	0.588	0.746	8.791
	3	8	0.651	0.195	0.553	0.188	0.255	0.356	1.107	1.704
miR326	1	9	0.268	0.159	0.478	0.045	0.052	0.135	0.187	1.534
	2	23	0.417	0.172	0.823	0.006	0.048	0.082	0.485	3.429
	3	8	0.1925	0.0525	0.1485	0.0328	0.0854	0.1343	0.3571	0.4245

**Table 8:** Mean expression of miRNA in cancer invasion.

Variable	Tissue Invasion	N	Mean	SE Mean	StDev	Variance	Minimum	Q1	Median	Q3	Maximum	Skewness
miR 101-3p	lymphovascular invasion	12	0.561	0.250	0.866	0.750	0.026	0.079	0.216	0.658	3.073	2.59
	muscular invasion	4	1.113	0.545	1.089	1.187	0.118	0.147	1.069	2.123	2.196	0.05
	organ metastasis	5	0.351	0.139	0.310	0.096	0.144	0.162	0.207	0.611	0.891	1.97
	serous invasion	17	0.2336	0.0690	0.2847	0.0810	0.0234	0.0798	0.1528	0.2419	1.0513	2.35
	submucosal invasion	2	0.1282	0.0485	0.0686	0.0047	0.0796	*	0.1282	*	0.1767	*
miR 106a-5p	lymphovascular invasion	12	1.547	0.687	2.381	5.667	0.050	0.349	0.778	1.873	8.791	2.99
	muscular invasion	4	0.720	0.169	0.338	0.114	0.345	0.397	0.714	1.050	1.108	0.08
	organ metastasis	5	0.4865	0.0980	0.2191	0.0480	0.2462	0.2632	0.5101	0.6981	0.7269	-0.11
	serous invasion	17	0.5824	0.0998	0.4115	0.1693	0.1450	0.2419	0.3894	0.9011	1.7041	1.28
	submucosal invasion	2	0.449	0.297	0.420	0.177	0.152	*	0.449	*	0.746	*
miR326	lymphovascular invasion	12	0.462	0.275	0.954	0.909	0.006	0.025	0.123	0.463	3.429	3.22
	muscular invasion	4	0.487	0.349	0.698	0.488	0.108	0.117	0.152	1.191	1.534	1.99
	organ metastasis	5	0.637	0.436	0.976	0.953	0.078	0.080	0.095	1.466	2.338	1.98
	serous invasion	17	0.1538	0.0459	0.1892	0.0358	0.0145	0.0522	0.0816	0.1922	0.7735	2.59
	submucosal invasion	2	0.1261	0.0784	0.1108	0.0123	0.0477	*	0.1261	*	0.2044	*

Note: “*” shows that there is missing data in that specific interval.

## Data Availability

The data presented in this study is available on request from the corresponding author.

## Abbreviations

WHO—World Health Organization; RA—rheumatoid arthritis; IBD—inflammatory bowel disease; LE—lupus erythematosus; CKD—chronic kidney disease.
